# Role of MUC5B during Group B Streptococcal Vaginal Colonization

**DOI:** 10.1128/mbio.00039-22

**Published:** 2022-03-24

**Authors:** Lindsey R. Burcham, Jade R. Bath, Caroline A. Werlang, Laurie M. Lyon, Naoko Liu, Christopher Evans, Katharina Ribbeck, Kelly S. Doran

**Affiliations:** a Department of Immunology and Microbiology, University of Colorado School of Medicine, Aurora, Colorado, USA; b Department of Biological Engineering, Massachusetts Institute of Technology, Cambridge, Massachusetts, USA; c Division of Pulmonary Sciences and Critical Care Medicine, University of Colorado School of Medicine, Aurora, Colorado, USA; University of Illinois at Chicago

**Keywords:** mucin, pili, vaginal colonization

## Abstract

The female reproductive tract (FRT) is a complex environment, rich in mucin glycoproteins that form a dense network on the surface of the underlying epithelia. Group B Streptococcus (GBS) asymptomatically colonizes 25–30% of healthy women, but during pregnancy can cause ascending infection *in utero* or be transmitted to the newborn during birth to cause invasive disease. Though the cervicovaginal mucosa is a natural site for GBS colonization, the specific interactions between GBS and mucins remain unknown. Here we demonstrate for the first time that MUC5B interacts directly with GBS and promotes barrier function by inhibiting both bacterial attachment to human epithelial cells and ascension from the vagina to the uterus in a murine model of GBS colonization. RNA sequencing analysis of GBS exposed to MUC5B identified 128 differentially expressed GBS genes, including upregulation of the pilus island-2b (PI-2b) locus. We subsequently show that PI-2b is important for GBS attachment to reproductive cells, binding to immobilized mucins, and vaginal colonization *in vivo*. Our results suggest that while MUC5B plays an important role in host defense, GBS upregulates pili in response to mucins to help promote persistence within the vaginal tract, illustrating the dynamic interplay between pathogen and host.

## OBSERVATION

The thick apical glycocalyx covering the epithelium of the FRT is primarily composed of mucin glycoproteins (1). Mucins can be attached to epithelial cell surfaces or released as gel-forming polymers into luminal spaces, and are characterized by large, filamentous protein domains extensively decorated with O-glycosidic linked short-chain glycans (2). In the FRT, the main cell surface associated mucins are MUC1 and MUC4, and the predominant secreted polymeric mucins are MUC5B and MUC5AC. Group B Streptococcus (GBS) is a Gram-positive bacterium that asymptomatically colonizes the FRT; however, during pregnancy GBS can cause intrauterine infection, premature rupture of membranes (PROM), and preterm birth (3, 4). GBS can also be transmitted to the fetus or newborn resulting in serious invasive infections such as pneumonia, sepsis, and meningitis (3). Though the cervicovaginal mucosa is a natural site for GBS colonization, the interactions between GBS and mucins have not been studied previously.

### MUC5B-GBS interactions.

**FIG 1:**
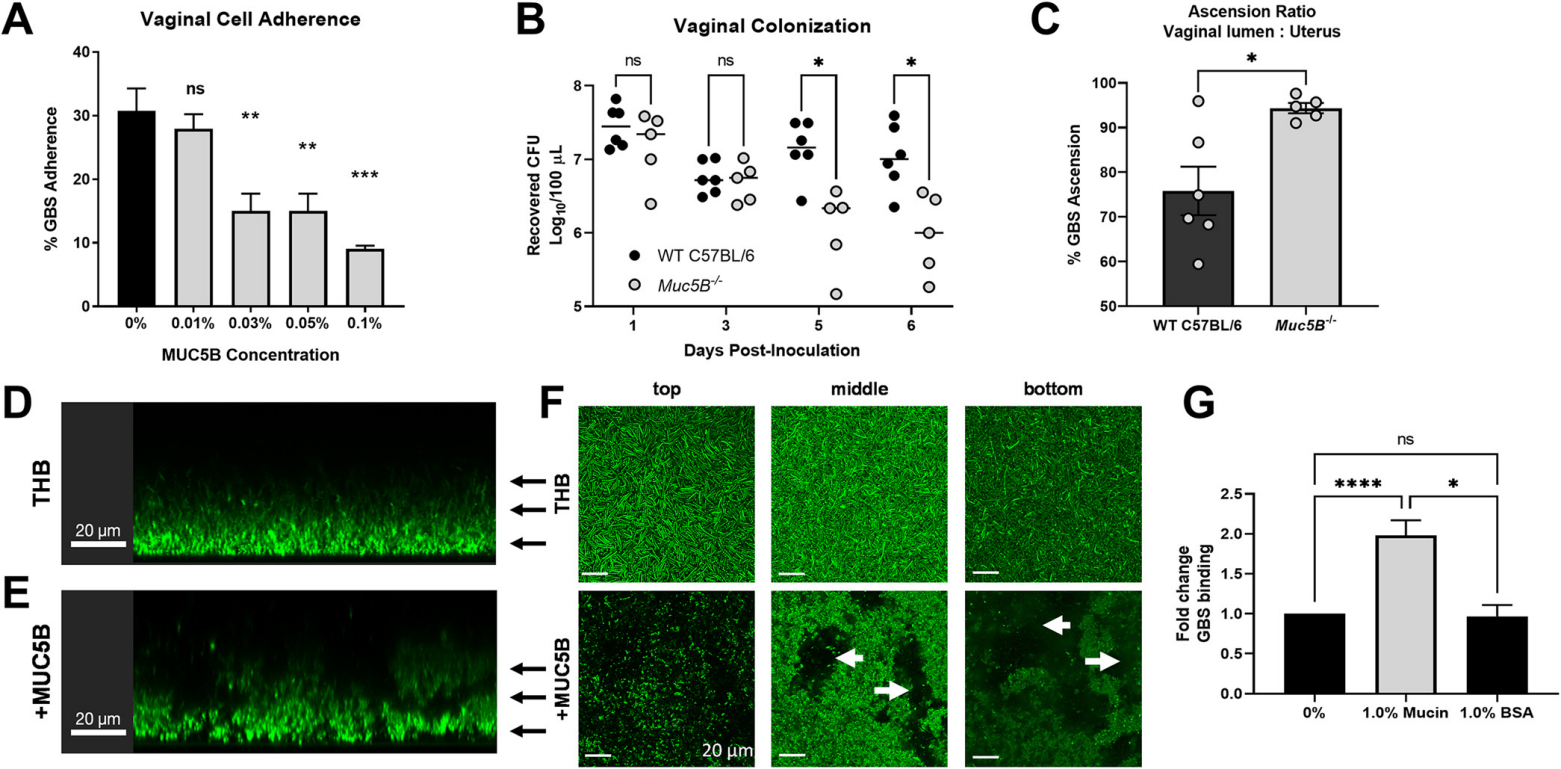
MUC5B impacts GBS spatial distribution, enhances GBS cell–cell interactions, and promotes vaginal colonization. (A) Adherence of GBS to VK2 cells with 0–0.1% MUC5B. Data represented as mean percent CFU recovered from inoculum. (B) Vaginal persistence of GBS in WT C57BL/6 and *Muc5B^−/−^* littermates (*n *= 6 WT, *n *= 5 *Muc5B^−/−^*, pooled data from two independent experiments). (C) Ascension ratio of GBS from the vaginal lumen to the uterus in C57BL/6 and *Muc5B^−/−^* mice 7 days postcolonization. (D–F) Confocal microscopy of GBS cultures grown in (D) Todd Hewitt Broth (THB) or (E) THB with MUC5B. Black arrows denote the vertical plane, with representative images taken from the (F) top, middle, and bottom of the culture. White arrows indicate furrows observed in the samples grown in MUC5B. (G) Binding of GBS to mucins or BSA. Statistical analyses were determined using (A) one-way ANOVA with Dunnett’s multiple comparison posttest, (B) two-way ANOVA with Sidak’s multiple comparison posttest, (C) Student’s unpaired, two-tailed *t* test, and (D) one-way ANOVA with Tukey’s multiple comparison posttest (G). Statistical significance was accepted when *p* < α, with α = 0.05; *, *P < *0.05; **, *P < *0.01; ***, *P < *0.001; ****, *P < *0.0001; ns, not significant.

To understand how mucins impact GBS colonization, we assessed GBS adherence to human vaginal epithelial cells in the presence of MUC5B and found that as low as 0.03% MUC5B inhibited bacterial attachment to vaginal cells (Fig. 1A, Fig. S1A in the supplemental material). To examine this interaction *in vivo*, we utilized a murine model of GBS vaginal colonization (5) and observed a significant decrease in recovered GBS from the vaginal lumen of *Muc5B*^−/−^ mice (6) compared to wild-type (WT) mice (Fig. 1B). Despite this, there was increased GBS ascension from the vaginal lumen to the uterus in *Muc5B^−/−^* mice, with a positive ascension Spearman correlation in *Muc5B^−/−^* mice (*P = *0.0167) that was absent in WT mice (*P = *0.1361) (Fig. 1C, Fig. S1B). These results suggest that mucins localize GBS in the vaginal lumen and limit GBS ascension. Future studies on how the microbiome and immunologic differences in *Muc5B^−/−^* mice may contribute to GBS ascension will be of interest.

We next visualized GBS in the presence of MUC5B by confocal microscopy. GBS grew homogenously through the column of control medium samples (Fig. 1D and F), while GBS grown in the presence of natively purified MUC5B mucins was heterogeneously distributed and formed aggregated structures with marked furrows throughout (Fig. 1E and F), suggesting that mucins agglutinate GBS, which could decrease interactions at the epithelial surface. Similar mechanisms of mucin-mediated agglutination have been characterized in oral Streptococci, where mucins aggregate bacteria to facilitate clearance by swallowing or enhancing phagocytosis (7). We further observed that GBS binds directly to immobilized mucins compared to untreated or 1% BSA control coated wells (Fig. 1G). While others have shown mucins can inhibit biofilm formation by some opportunistic pathogens (8–10), we observed that GBS grown in the presence of MUC5B formed larger aggregates compared to those grown in control medium alone and the addition of mucins did not impact bacterial growth (Fig. S1C–F).

### MUC5B modulates the GBS transcriptome.

**FIG 2:**
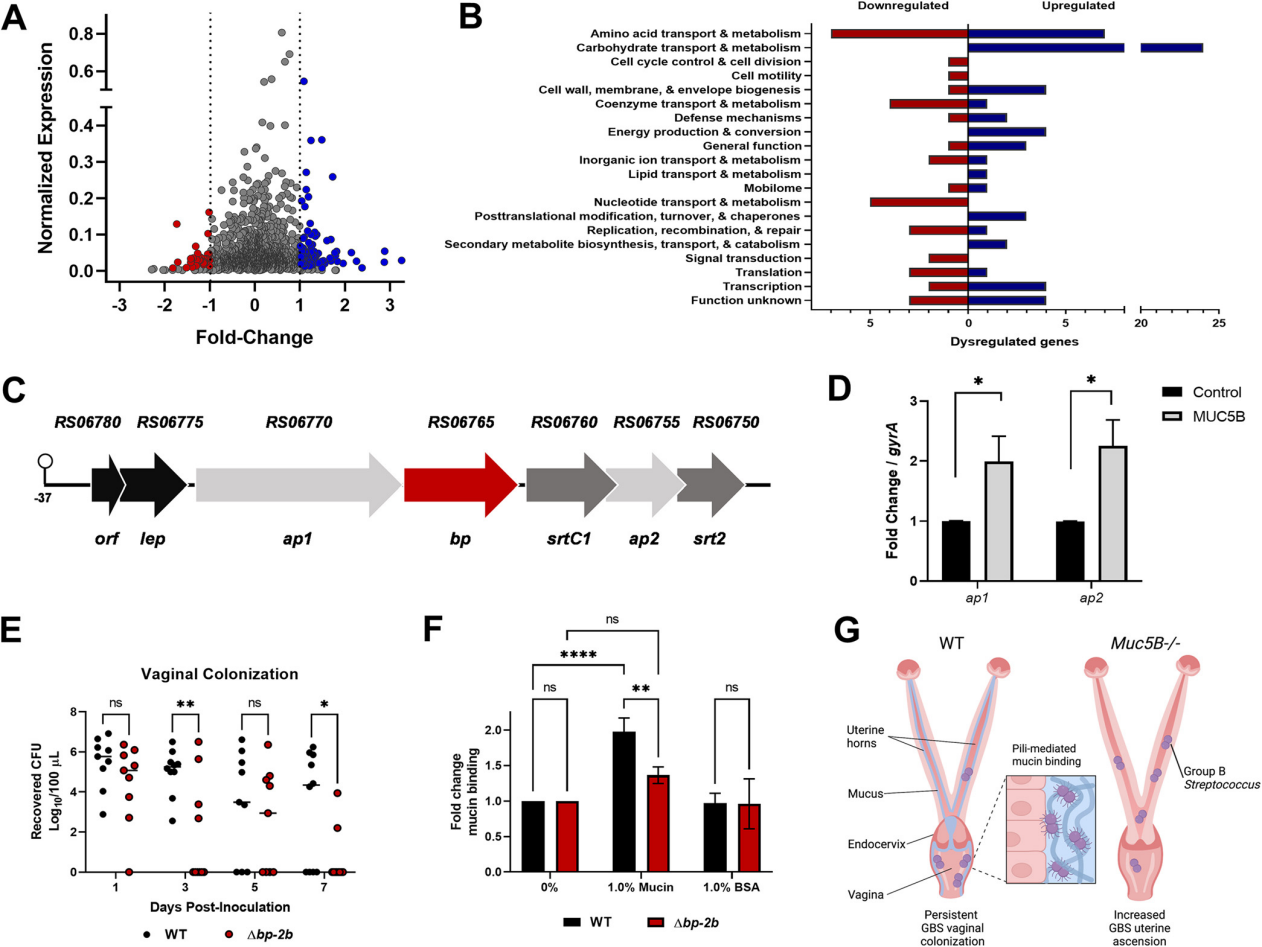
Pilus island-2b (PI-2b) is upregulated in the presence of mucin and contributes to host interactions. (A) Volcano plot indicates combined analysis from DESeq2 and EdgeR. Red (downregulated), blue (upregulated), and gray (unchanged) represent differential gene expression following MUC5B exposure (*P < *0.05, fold change >±2). (B) COG assignments determined for differentially expressed genes using EggNOG 5.0. (C) Diagram of the GBS PI-2b locus with the previously described promoter indicated at −37 bp (21). The gene shown in red encodes the major pilin backbone, genes shown in light gray represent the genes encoding the accessory pilus proteins, and the genes in dark gray represent the anchoring sortases. (D) Expression of *ap1* and *ap2* were assessed by qRT-PCR to confirm upregulation of the PI-2b locus observed in RNA sequencing analysis. (E) Persistence of WT and Δ*bp-2b* in competition in CD-1 vaginal colonization (*n *= 10). (F) Binding of WT and Δ*bp-2b* to immobilized mucins. (G) Model created with BioRender.com showing reproductive tract mucins as both a barrier against uterine ascension and a substrate for GBS pilus-mediated interactions to promote persistence within the vaginal lumen. Statistical analyses were determined using Student’s unpaired, two-tailed *t* test (D) and two-way ANOVA with Sidak’s multiple-comparison test (E and F). Statistical significance was accepted when *p* < α, with α = 0.05; *, *P < *0.05; **, *P < *0.01; ****, *P < *0.0001; ns, not significant.

To determine the GBS response to mucins, we performed RNA sequencing analyses of GBS grown with MUC5B and identified 128 differentially expressed genes including 87 upregulated and 41 downregulated genes (Fig. 2A, Table S1). Clusters of orthologous proteins (COGs) were identified for 63 upregulated and 37 downregulated genes (Fig. 2B). The most abundant upregulated COGs were involved in carbohydrate transport/metabolism (24/63, 38%), suggesting signaling could occur via the carbohydrate moieties on the mucin polymers. Many bacterial species possess mucolytic enzymes that can release and metabolize sugars from the mucin backbone (11, 12), and examples have been primarily described in organisms isolated from the human gastrointestinal tract (13, 14). While GBS is known to possess *trans*-sialidases that could contribute to this process, they have not been studied in this context (15). In studies with other opportunistic pathogens, mucin exposure suppressed virulence traits (8–10); however, our analyses revealed an upregulation of GBS virulence factors including the type VII secretion system (T7SS) and the pilus island-2b (PI-2b). The GBS T7SS was recently identified and shown to contribute to invasive disease (16), although its role in vaginal colonization remains to be determined.

### PI-2b contributes to mucin interactions and vaginal colonization.

All GBS strains express one or two pili encoded by three different pilus islands, PI-1, PI-2a, or PI-2b (17). The GBS COH1 strain used here is a serotype III, sequence type-17 and encodes pilus islands PI-1 and PI-2b, but only PI-2b was shown to be required for interaction with brain endothelium and lung epithelial cells, as well as the development of invasive disease (17, 18). To characterize the role of PI-2b in GBS mucosal interactions within the FRT, we confirmed differential expression of genes within the PI-2b locus (Fig. 2C) by qRT-PCR following incubation with MUC5B (Fig. 2D). We then determined that GBS lacking the main backbone pilin protein (Δ*bp-2b*) was less adherent than WT GBS to both squamous vaginal epithelial cells and columnar endocervical epithelial cells, and this phenotype was complemented by expression of the *bp-2b* gene in the Δ*bp-2b* mutant strain (Δ*bp-2b*+*bp-2b*) (Fig. S1G, H). To examine the role of PI-2b *in vivo*, mice were inoculated intravaginally with GBS WT and Δ*bp-2b* strains, and bacterial burden within the lumen was monitored over time. We observed a fitness defect in the Δ*bp-2b* mutant in the vaginal tract compared to WT GBS (Fig. 2E). We also observed a significant reduction in mucin binding by the Δ*bp-2b* mutant (Fig. 2F), which is consistent with other studies showing bacterial pili can promote interactions with colonic mucus and salivary glycoproteins (19, 20). These data suggest a key role for PI-2b in facilitating GBS attachment to host mucins and the reproductive epithelium, and persistence within the vaginal mucosa *in vivo*. Future studies examining the role of PI-2b in GBS tissue colonization and ascension are warranted.

### Conclusions.

Here we describe for the first time the dynamic interplay between GBS and host mucins in the FRT. MUC5B induced GBS agglutination, prevented bacterial attachment to epithelial cells, concentrated GBS in the vaginal lumen, and served as a barrier against ascending infection (Fig. 2G). MUC5B also induced GBS virulence factors such as PI-2b that contribute to host cell attachment and FRT colonization. As protein–carbohydrate interactions typically mediate the first step in pathogen colonization, we hypothesize that GBS pili normally bind carbohydrate moieties on the epithelium, which could be outcompeted by glycosylated mucins in this environment, though this warrants further investigation. These initial observations provide a platform for future studies to understand the mechanisms of GBS-reproductive mucosal interactions that could lead to novel interventions to improve maternal–fetal health, reduce adverse pregnancy outcomes, and limit transmission to the newborn.

10.1128/mbio.00039-22.1 FIG S1 (A) Adherence of GBS to VK2 cells with 0.1–0.5% MUC5B. Data are represented as a mean percent CFU recovered from the inoculum. (B) Spearman correlation between GBS recovered from the vaginal lumen and the uterine tissues of WT and *Muc5B^−/−^* mice. COH1 or GFP-expressing COH1 were grown for 18 h in media alone or with MUC5B to assess biofilm formation. Biofilms were rinsed and (C) stained with crystal violet to assess optical density (OD_595nm_) or (D) fixed and imaged by fluorescence microscopy using a BZ-X710 Keyence microscope. Growth of GBS with and without MUC5B was assessed by (E) CFU quantification or (F) optical density (OD_600nm_). GBS COH1 grown +/– MUC5B CFU data are displayed as average from *n *= 3 biological replicates. Adherence of WT, Δ*bp-2b*, and Δ*bp-2b+bp-2b* to (G) vaginal and (H) endocervical (End1) epithelial cells. Data are represented as a mean percent CFU recovered from the inoculum of three independent experiments. Statistical analyses were determined using one-way ANOVA with Dunnett’s multiple comparison posttest (A), Student’s unpaired, two-tailed *t* test (C, E), and one-way ANOVA with Tukey’s multiple comparison posttest (G, H). Statistical significance was accepted when *p* < α, with α = 0.05; *, *P < *0.05; **, *P < *0.01; ***, *P < *0.001; ****, *P < *0.0001; ns, not significant. Download FIG S1, PDF file, 0.2 MB. Copyright © 2022 Burcham et al. 2022 Burcham et al. https://creativecommons.org/licenses/by/4.0/ This content is distributed under the terms of the Creative Commons Attribution 4.0 International license.

10.1128/mbio.00039-22.2 TABLE S1 RNA sequencing data from GBS COH1 +/– 0.3% MUC5B. Download Table S1, XLSX file, 0.4 MB. Copyright © 2022 Burcham et al. 2022 Burcham et al. https://creativecommons.org/licenses/by/4.0/ This content is distributed under the terms of the Creative Commons Attribution 4.0 International license.

10.1128/mbio.00039-22.3 TEXT S1 Supplemental methods. Download Text S1, DOCX file, 0.03 MB. Copyright © 2022 Burcham et al. 2022 Burcham et al. https://creativecommons.org/licenses/by/4.0/ This content is distributed under the terms of the Creative Commons Attribution 4.0 International license.
