# Supplementary material for: Role of MUC5B during Group B Streptococcal Vaginal Colonization
Source: mBio. 2022 Mar 24;13(2):e00039-22. doi: 10.1128/mbio.00039-22 (PMC9040740; doi:10.1128/mbio.00039-22)
Supplement: FIG S1 [file mbio.00039-22-sf001.pdf]

Supplemental Materials

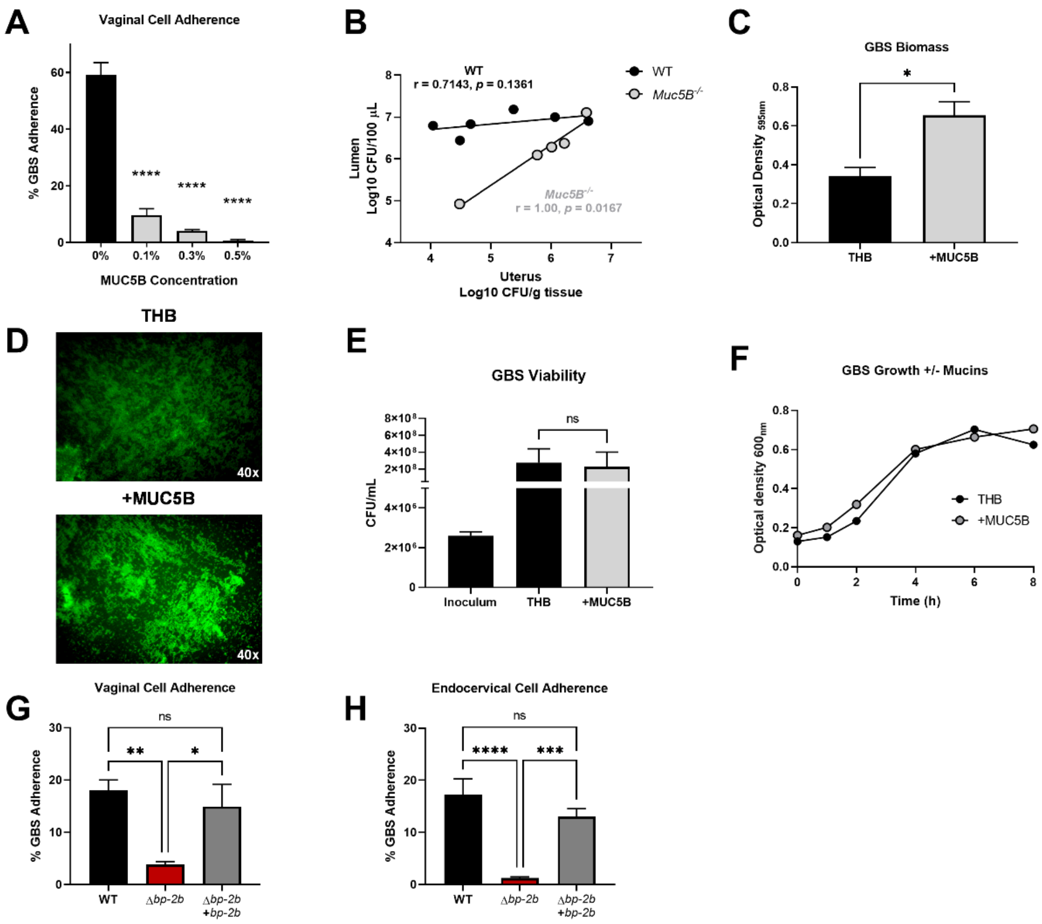

Supplemental Figure 1. A) Adherence of GBS to VK2 cells with 0.1-0.5% MUC5B. Data are represented as a mean percent CFU recovered from the inoculum. **B)** Spearman correlation between GBS recovered from the vaginal lumen and the uterine tissues of WT and *Muc5B*$^{-/-}$ mice. COH1 or GFP-expressing COH1 were grown for 18h in media alone or with MUC5B to assess biofilm formation. Biofilms were rinsed and **C)** stained with crystal violet to assess optical density (OD_{595nm}) or **D)** fixed and imaged by fluorescence microscopy using a BZ-X710 Keyence microscope. Growth of GBS with and without MUC5B was assessed by **E)** CFU quantification or **F)** optical density (OD_{600nm}). GBS COH1 grown +/- MUC5B CFU data are displayed as average from n=3 biological replicates. Adherence of WT, Δ*bp-2b*, and Δ*bp-2b+bp-2b* to **G)** vaginal and **H)** endocervical (End1) epithelial cells. Data are represented as a mean percent CFU recovered from the inoculum of three independent experiments. Statistical analyses were determined using One-way ANOVA with Dunnett's multiple comparison post-test **(A)**, Student's unpaired, two-tailed t-test **(C, E)**, and One-way ANOVA with Tukey's multiple comparison post-test **(G, H)**. Statistical significance was accepted when $p < \alpha$, with $\alpha = 0.05$, * $p<0.05$, ** $p<0.01$ *** $p<0.001$, **** $p<0.0001$, and ns = not significant.
