# Supplementary material for: Role of MUC5B during Group B Streptococcal Vaginal Colonization
Source: mBio. 2022 Mar 24;13(2):e00039-22. doi: 10.1128/mbio.00039-22 (PMC9040740; doi:10.1128/mbio.00039-22)
Supplement: TEXT S1 [file mbio.00039-22-s0001.docx]

**Supplemental Methods**

***Study Approval.*** All experiments using pathogens were performed under approval of the Institutional Biosafety Committee (#17-025). Animal experiments were conducted under the approval of the Institutional Animal Care and Use Committee (#00316) at the University of Colorado Anschutz Medical Campus and performed using accepted veterinary standards. Protocols using saliva samples from human participants were approved by the Massachusetts Institute of Technology’s Committee on the Use of Humans as Experimental Subjects (#1312006096).

***Bacterial Strains and Growth Conditions.*** *Streptococcus agalactiae* (GBS) isolates COH1 (serotype III), pilus island-2b mutant strain (Δ*bp-2b*), and complemented strain (Δ*bp-2b*­+*bp-2b* were cultured in Todd-Hewitt Broth (THB) at 37°C. The Δ*bp-2b* mutant strain and complement were generated as previously described (1).

***Confocal Microscopy.*** Overnight cultures of GBS COH1 were diluted 1:20 into fresh THB media and cultured with or without 0.3% purified-MUC5B in a glass-bottom 96-well plate for 24 hours at 37°C. Before imaging, cells were stained for 15 minutes with 5 μM SYTO 9 (ThermoFisher). Plates were examined via microscopy to determine the spatial distribution of cells in each well. Image acquisition was performed using a confocal laser scanning microscope (LSM 800; Zeiss) equipped with a 63×/1.4 NA oil immersion or a 100×/1.4 NA oil immersion objective. Images were analyzed with Zeiss ZEN imaging software (Thornwood, NY, USA). The excitation wavelength for SYTO 9 was 488 nm. At least five stacks were recorded for each well and at least three independent wells were analyzed for each condition. Images were acquired with a step size of 0.5 μm. 3D images of cultures were created with IMARIS 9.7.2 (Bitplane, Switzerland).

***Aggregation analyses.*** Overnight cultures of COH1 or GFP-expressing COH1 were diluted 1:50 into fresh KSF media and cultured with or without added 0.3% purified-MUC5B for 18 hours at 37°C. For crystal violet assays, GBS aggregates were washed after incubation one time with PBS and stained with 0.5% crystal violet for 15 minutes. The crystal violet was then removed, wells were rinsed two times with PBS, air dried, and the crystal violet solubilized with 95% ethanol. The optical density was collected at a wavelength of 595 nm. Experiments were performed in technical replicates for three independent experiments. Data are displayed as the mean of the three independent experiments. For fluorescence microscopy, aggregates of GFP-expressing GBS were cultured as above on chamber-well slides (Nunc). Following the 18-hour incubation, aggregates were rinsed one time with PBS, fixed with Fluoroshield™ (Sigma), and a glass coverslip was attached to the slide. Aggregates were imaged in the GFP-channel using a Keyence BZ-X710 fluorescence microscope.

***Mucin binding assays.*** Overnight cultures of WT COH1 or Δ*bp-2b* strain were inoculated into 96 wells coated with control KSF media, 1% mucins, or 1% BSA solubilized in KSF media. Plates were incubated for 2 hours at 37°C. After incubation, wells were rinsed one time with PBS and stained with 0.5% crystal violet for 15 minutes. The crystal violet was then removed, plates were rinsed two times with PBS, dried, and the crystal violet solubilized with 95% ethanol. The optical density was collected at a wavelength of 595 nm. Mucin binding experiments were performed in technical replicates for three independent experiments. Data are displayed as the mean of the three independent experiments, with the control wells (KSF coated) set to 1 fold.

***Mice***. All mice were housed in pathogen-free, biosafety level-2 animal facilities in accordance with the Institutional Animal Care and Use Committee of the University of Colorado (protocol #00316). C57BL/6 *Muc5B*^+/-^ were generated as previously described (2) and heterogeneous males and females were bred to generate female *Muc5B*^+/+^ littermate controls and *Muc5B*^-/-^ (RRID: MGI:5576339) for use in experiments. CD-1 mice for WT and Δ*bp-2b* GBS challenges were purchased from Charles River.

***Murine model of GBS vaginal colonization.*** We utilized a mouse model of vaginal colonization as previously described, where female eight-week-old C57BL/6, C57BL/6 *Muc5B*^-/-^, and CD-1 mice were injected intraperitoneally with β-estradiol 0.5 mg/100 μL. Twenty-four hours later, mice were inoculated intravaginally with 1x10^7^ CFU GBS. Mice were swabbed daily and recovered bacteria were plated on CHROMagar selective and differential media to enumerate bacterial burden over time. To determine bacterial load in tissues, mice were euthanized and reproductive tissues were harvested. Tissues were homogenized in sterile PBS and plated on CHROMagar to quantify GBS CFU burden.

***RNA Sequencing.*** Cultures of GBS COH1 were grown in triplicate to OD_600nm_ 0.4 and 1x10^6^ CFU/mL were diluted in KSF media +/- 0.3% MUC5B. Cultures were incubated for 1 hour and lysed by beating with 0.1 mM zirconia beads (Biospec Products) for a total of 3 minutes. RNA was isolated from lysates using the Macherey-Nagel NucleoSpin^®^ RNA kit (Macherey-Nagel) per manufacturer’s instructions. Illumina cDNA libraries were prepared and sequenced at the Broad Institute of MIT and Harvard Microbial “Omics” Core as previously described (3, 4). Approximately 1 μg of total RNA was fragmented, depleted of genomic DNA, dephosphorylated, and ligated to DNA adapters carrying 5′-AN8-3′ barcodes of known sequence with a 5′ phosphate and a 3′ blocking group. Barcoded RNAs were pooled and depleted of rRNA using a RiboZero rRNA depletion kit (Epicenter). Pools of barcoded RNAs were converted to Illumina cDNA libraries via reverse transcription of the RNA using a primer designed to be specific to the constant region of the barcoded adapter with addition of an adapter to the 3′ end of the cDNA by template switching using SMARTScribe reverse transcriptase (Clontech) as described previously (3) and PCR amplification using primers whose 5′ ends target the constant regions of the 3′ or 5′ adapters and whose 3′ ends contain the full Illumina P5 or P7 sequences. cDNA libraries were sequenced on an Illumina NextSeq 500 platform to generate paired end reads.

Coding sequences from DESeq2 and EdgeR analyses were compared, and transcripts with an adjusted p-value <0.05 by both analyses and a mean log 2-fold change ±1 were considered significant to reduce procedural bias. Mean transcript counts were normalized between the two methods by relative abundance transformation as previously described (4). Transcripts were annotated using GenBank, accession [NZ_HG939456.1](https://www.ncbi.nlm.nih.gov/nuccore/NZ_HG939456.1) and COGs were assigned to dysregulated genes using eggnog 5.0 (5). Volcano plots were generated using the GraphPad Prism version 9.2.

***Data availability.*** Sequencing reads from the RNA sequencing analyses are available in the European Nucleotide Archive (ENA) under the accession number (PRJEB49122).

***RNA Extraction and Quantitative Reverse Transcriptase PCR (qRT-PCR).*** GBS were grown to mid-logarithmic phase (OD_600nm_ 0.4) in THB media, cells were rinsed and resuspended in KSF media with or without 0.3% MUC5B and incubated for 1 hour at 37°C. Following incubation, bacteria were centrifuged at 5000 rpm for 5 minutes, total RNA was extracted as described above with the addition of a 30-minute on-column DNase treatment. For qRT-PCR analyses, cDNA synthesis was performed with 500 ng RNA template (Quanta Biosciences) per manufacturers’ instructions. Fold changes were calculated by ΔΔCT analysis with *gyrA* serving as an internal housekeeping control. Primers used in this study include: *gyrA* F: agcacaaaaacgtggaggac, *gyrA* R: acgatagggaggcctttagc, *ap1 (SAN1516)* F: ttccctttgtctgatgtgttact, *ap1* *(SAN1516)* R: taccggctgaaggtattgttg, *ap2 (SAN1519)* F: gtcaccagccctgaatacatag, *ap2* *(SAN1519)* R: acttatgatgctaccggcttac.

***Cell lines and adherence assays.*** Human VK2 vaginal epithelial (ATCC CRL-2616) and End1 endocervical epithelial (ATCC CRL-2615) cell lines were cultured in keratinocyte serum-free medium (KSFM) supplemented with 0.1 ng/mL human recombinant epidermal growth factor (Gibco) and 0.05 mg/mL bovine pituitary extract (Gibco) at 37°C with 5% CO_2_. Adherence assays were performed as previously described (4). Briefly, bacteria were grown to mid-logarithmic phase and cell monolayers were infected at a multiplicity of infection (MOI) of 1. Following a 30-minute incubation, cells were rinsed 5 times with PBS, released with 0.25% trypsin-EDTA (Gibco), and lysed with 0.025% Triton-X 100. Cell lysates were serially diluted and plated on TSA to quantitate bacterial CFU. Experiments were performed in triplicate wells in three independent experiments. Data are displayed as the average of mean adherence from *n*=3 independent experiments.For adherence experiments with added mucins, experiments were performed in 96-well plates and purified MUC5B was added at concentrations ranging from 0-0.5% prior to GBS infections. Data shown are mean adherence from individual experiments.

**Supplemental References**

1. Lazzarin M, Mu R, Fabbrini M, Ghezzo C, Rinaudo CD, Doran KS, Margarit I. 2017. Contribution of pilus type 2b to invasive disease caused by a *Streptococcus agalactiae* ST-17 strain. BMC Microbiology 17:148.

2. Roy MG, Livraghi-Butrico A, Fletcher AA, McElwee MM, Evans SE, Boerner RM, Alexander SN, Bellinghausen LK, Song AS, Petrova YM. 2014. Muc5b is required for airway defence. Nature 505:412-416.

3. Deng L, Mu R, Weston TA, Spencer BL, Liles RP, Doran KS. 2018. Characterization of a Two-Component System Transcriptional Regulator, LtdR, That Impacts Group B Streptococcal Colonization and Disease. Infect Immun 86.

4. Spencer BL, Deng L, Patras KA, Burcham ZM, Sanches GF, Nagao PE, Doran KS. 2019. Cas9 Contributes to Group B Streptococcal Colonization and Disease. Frontiers in microbiology 10:1930-1930.

5. Huerta-Cepas J, Szklarczyk D, Heller D, Hernández-Plaza A, Forslund SK, Cook H, Mende DR, Letunic I, Rattei T, Jensen Lars J, von Mering C, Bork P. 2018. eggNOG 5.0: a hierarchical, functionally and phylogenetically annotated orthology resource based on 5090 organisms and 2502 viruses. Nucleic Acids Research 47:D309-D314.
